# Electrochemical detection of miRNA using commercial and hand‐made screen‐printed electrodes: liquid biopsy for cancer management as case of study

**DOI:** 10.1002/open.202300203

**Published:** 2024-02-09

**Authors:** Ada Raucci, Wanda Cimmino, Sabrina Romanò, Sima Singh, Nicola Normanno, Federico Polo, Stefano Cinti

**Affiliations:** ^1^ Department of Pharmacy University of Naples Federico II Via Domenico Montesano 9 80131 Naples Italy; ^2^ Cell Biology and Biotherapy Unit Istituto Nazionale Tumori (IRCCS) Fondazione Pascale Via Mariano Semmola 53 80131 Naples Italy; ^3^ Department of Molecular Sciences and Nanosystems Ca' Foscari University of Venice Via Torino 155 30172 Venice Italy

**Keywords:** Electroanalysis, Liquid biopsy, miRNA, Paper-based, Screen-printed electrodes

## Abstract

The growth of liquid biopsy, i. e., the possibility of obtaining health information by analysing circulating species (nucleic acids, cells, proteins, and vesicles) in peripheric biofluids, is pushing the field of sensors and biosensors beyond the limit to provide decentralised solutions for nonspecialists. In particular, among all the circulating species that can be adopted in managing cancer evolution, both for diagnostic and prognostic applications, microRNAs have been highly studied and detected. The development of electrochemical devices is particularly relevant for liquid biopsy purposes, and the screen‐printed electrodes (SPEs) represent one of the building blocks for producing novel portable devices. In this work, we have taken miR‐2115‐3p as model target (it is related to lung cancer), and we have developed a biosensor by exploiting the use of a complementary DNA probe modified with methylene blue as redox mediator. In particular, the chosen sensing architecture was applied to serum measurements of the selected miRNA, obtaining a detection limit within the low nanomolar range; in addition, various platforms were interrogated, namely commercial and hand‐made SPEs, with the aim of providing the reader with some insights about the optimal platform to be used by considering both the cost and the analytical performance.

## Graphical abstract

Screen‐printed electrodes are able to offer potent tools for detecting miRNAs in liquid biopsy: commercial or handmade?

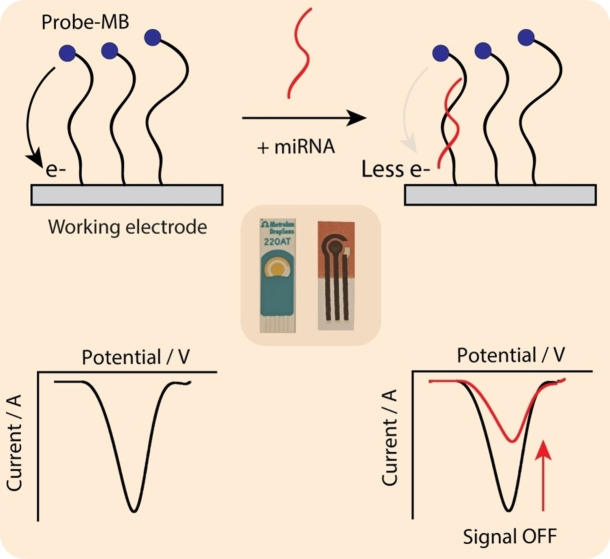

## Introduction

Lung cancer leads to almost 1.8 million deaths annually worldwide and is the leading cause of cancer‐related death in both men and women worldwide.^[1]^ Approximately half of people who fight lung cancer die within one year after being diagnosed. The survival rate beyond five years is also only 19 %.^[2]^ 85 % of lung cancers are classified as non‐small cell lung cancers (NSCLC). Lymph node (LN) metastasis is among the important metastasis pathways in NSCLC and is an essential clinical indicator. This also reduces the 5‐year survival rate of patients from 75 % to just 20 %.^[3]^

However, with reference to present oncology immune checkpoint inhibitors (ICIs) such as anti‐cytotoxic T lymphocyte associated protein 4, (CTLA‐4) and anti‐programmed cell death 1 (PD‐1) are better at treating cancer than traditional chemotherapy. Response rates in NSCLC using anti‐PD‐1 range from 17 to 21 %^[4]^ However, this approach can treat or benefit only a fraction of NSCLC patients. To enhance the effectiveness of ICI therapy and improve patient survival dependent on immunotherapy, it is crucial to have reliable biomarkers that can accurately predict treatment response. Consequently, it is valuable to pinpoint specific markers that could facilitate earlier diagnosis and monitoring, preferably through noninvasive means. These parameters have the potential to help guide therapeutic decision‐making for patients, thereby reducing the likelihood of subsequent metastases.^[5]^

Liquid biopsy methods have been developed that allow continuous follow‐up through the sampling of blood‐based biomarkers, providing the opportunity to improve patient care with simple and rapidly available assays.^[6]^ Some of such circulating biomarkers with notable potential include CTC, ctDNA, ctRNA, miRNAs, lncRNAs, EVs and antibodies that have shown robust diagnostic, therapeutic and prognostic potential.[^7^, ^8^] Among these, circulating miRNA have drawn a lot of attention from researchers toward the development of a feasible detection platform with clinical utility in predicting tumours, diagnosis, and response treatment.^[9]^

For example, Rajakumar et al. analysed blood samples from 344 patients diagnosed with stage four NSCLC, showing that a characteristic profile of myeloid cell synthesised miRNA could serve as a predictor of survival after immunotherapies treatment. A new panel named ′the miRisk Score′, which comprises 5 microRNAs (miR‐2115‐3p, miR‐218‐5p, miR‐224‐5p, miR‐4676‐3p, miR‐6503‐5p) from mainly neutrophils, platelets, and monocytes was found to score low from patients with positive response to immunotherapy. The miRisk score was a stronger predictor of overall survival and responded to PD−I inhibitor monotherapy.^[10]^ Furthermore, the role of miR‐2115‐3p in the pathogenesis and prognosis of different types of cancer, such as triple‐negative breast cancer (TNBC),^[11]^ and brain metastases^[12]^ has been described.

miR‐2115‐3p is a pivotal molecular marker in recent immunotherapy advances, playing a key role in analyzing treatment responses. Its significance lies in informing decisions on immunotherapeutic interventions and enhancing individual responses. Detecting miR‐2115‐3p is crucial for a nuanced understanding of treatment dynamics, offering insights into disease progression and therapeutic efficacy. To date it is mostly detected by reverse transcription‐quantitative polymerase chain reaction (RT‐qPCR)^[12]^ and microarray hybridization^[13]^ techniques. In designing this test, researchers faced few competing challenges. Thus, so far these challenges have prevented their successful translation to clinical use due to low sensitivity or the limitations arising from the highly specialised devices and instruments needed. An alternative methodology is needed.

Based on this, here we propose a methodology involving electrochemical technology for the purpose of directing research efforts toward non‐invasive and simple early detection of miRNAs, allowing for timely examination and treatment intervention. Although electrochemical techniques have shown great potential to analyse miRNA for other diseases/cancers,[^14^, ^15^] none of the previous studies have dealt with applications to detect miR‐2115‐3p. This method enables efficient miR‐2115‐3p analysis with minimal samples, quick results, low cost, and on a point‐of‐care platform. Developing point‐of‐care tests (PoCT) meeting REASSURED criteria is vital for enhancing cancer management globally, especially in low‐income countries.^[16]^ Miniaturized electrochemical sensors, harnessing the benefits of microfabrication and employing hybrid nanoprobes, have proven effective in detecting clinically relevant molecules such as exosomes, proteins, nucleic acids, and enzymes. Identification of circulating nucleic acid sequences on electrochemical strips is primarily achieved through chemically bound complementary DNA, PNA, or RNA single strands on the electrode surface.[^17^, ^18^]

These probes can be combined with different sensing architectures: signal on and signal off which both rely on high selective target/probe hybridization.^[19]^ For signal off strategies, a redox mediator (i. e., methylene blue) is covalently attached to the recognition probe, while the signal on device exploited the use of an external redox mediator (i. e., ruthenium examine) which resulted electrostatically attached to the probe target more than the the probe alone.^[20]^ It is worth noting that both strategies were recently combined with electrochemical strips of electrochemical strips of different types of of screen‐printed electrodes (SPEs) coupled with portable potentiostats optimal in terms of user‐friendly, rapidity,, and real‐time connectivity.^[14]^ Among the strips tested, commercial gold SPEs and graphite SPEs modified with gold nanoparticles (AuNPs) showed promising results and several advantages, such as scalable production, ease of functionalisation (the thiolated probe is directly immobilised at the surface via Au−S chemistry) and satisfactory analytical performance.

Based on the advantages of the signal off architecture, the present paper deals with the comparative potential benefits and limitations of these electrochemical strips in the context of miR‐2115‐3p sensing. By systematically evaluating both the advantages and limitations, this work contributes to a more informed and sustainable use of paper‐based electrochemical strips in the field of miRNA sensing, fostering advances in diagnostic technologies with a focus on biological fluid analysis. In this study, we monitored miR‐2115‐3p using a biosensor modified with anti‐miRNA ssDNA and labelled with methylene blue (MB). The detection method involved monitoring MB′s electrochemical signal decrease in phosphate buffer and serum samples, all the experimental parameters were optimised and the performance of all the SPE‐based platforms was compared, in order to provide the reader with a clear indication regarding the choice of the platform to be used in sensing miRNA.

## Experimental Section

### Material and instrumentation

To evaluate the sensing architectures, two varieties of SPEs were used: gold SPEs (code: 220AT) sourced from Metrohm and carbon paper‐based screen printed electrodes produced by a manual printing method, as detailed in previous research. [24] Briefly, office paper (Fabriano Copy2, 80 g/m^2^) was used to screen‐print the electrodes. The testing area was obtained with the use of solid‐ink printer, and the curing of 100 °C allowed the solid ink (i. e. wax) to penetrate the paper and to create a hydrophobic area. Ag/AgCl ink (SunChemical, Code: C2130809D5, USA) has been used to obtain the reference electrode, while the carbon ink (SunChemical, C2030519P4, USA), has been adopted to realize the working and counter electrodes. Each of the conductive inks was manually screen‐printed using a squeegee and a mask. After the Ag/AgCl ink was printed, the electrodes were cured in the oven at 70 °C for 30 minutes. The same curing procedure was applied following the carbon screen‐printing. 4 mm was the diameter of the working electrode. To compare the two types of SPEs, the latter was modified with a dispersion of AuNPs that have been synthesised in house using gold chloride, sodium citrate, and sodium borohydride as precursors.^[25]^ Both the miRNA target, the anti‐miRNA DNA probe (modified with SH and methylene blue) and interference sequences were purchased from Metabion via Carlo Erba (Italy). The study sequence object was the following: target (5′‐CAUCAGAAUUCAUGGAGGCUAG‐3′), probe (5′‐ SH−C6‐CTAGCCTCCATGAATTCTGATG‐MB‐3′, and interference sequences (miR‐652‐5p 5′‐CAACCCUAGGAGGGGGUGCCAUUC‐3′, miR‐29‐a 5’‐TAGCACCATCTG AAATCGGTT‐3’, and miR‐21 5’‐UAGCUUAUCAGACUGAUGUUGA‐3’). All the reagents, including sodium chloride, 6‐Mercapto‐1‐hexanol, gold chloride, sodium citrate, sodium dihydrogen phosphate hydrate sodium hydrogen phosphate, tris(2‐carboxyethyl) phosphine and human serum were obtained from Sigma‐Aldrich (Italy). To conduct the electrochemical analysis, a multi‐EmStat (Palmsens, Netherlands) was used: this potentiostat allows one to perform up to eight measures simultaneously, using eight parallel channels.

### Sensor development and mechanism of recognition

**Figure 1:**
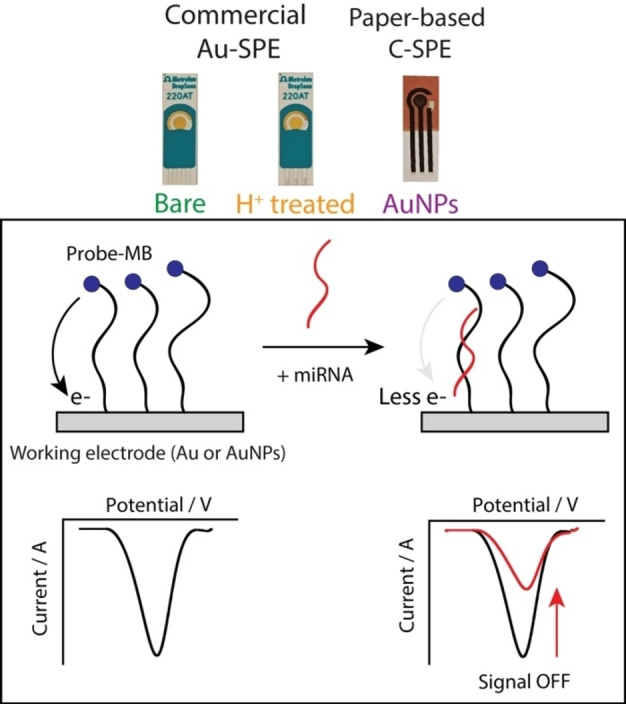
In the upper part of the figure the three types of SPEs used for this study are reported, namely commercial bare Au‐SPE (left), acidic treated commercial aAu‐SPE (center) and AuNP‐modified paper‐based carbon SPE. In the lower part, the mechanism of electrochemical sensing of the presence of miRNA is reported through the development of a signal OFF platform, which corresponds to a decrease of current when the concentration of target increases.

The sensor modification was based on a well‐established protocol published by Plaxco *et al*.^[18]^ Three types of SPE were modified, namely bare gold commercial, commercial gold treated and AuNP‐modified paper carbon electrode. Since the SPE was selected, three steps were performed: 1) reduction of the 100 μM probe in phosphate buffer (pH=7.4) in the presence of 10 mM tris(2‐carboxyethyl) phosphine) for 1 h at room temperature; 2) immobilisation of the reduced probe (at the chosen concentration) on the Au‐based surface by drop casting 20 μL onto the working electrode area for 1 h at room temperature in a humid chamber, and subsequently the SPEs were rinsed with distilled water; 3) incubation by drop casting 20 μL mercapto hexanol for 1.5 h at room temperature in a humid chamber, and subsequently the SPEs were rinsed with distilled water, and covered with phosphate buffer until its use. The mechanism of recognition of the miRNA target is based on hybridisation with the immobilised probe. When the target is absent, the MB is allowed to exchange electrons at the surface of the SPE, thus generating a current signal through voltametric analysis (current blank). When the target is present, the formation of the formation of the duplex limits the mobility of MB and results in decreased electron transfer to the surface of the SPE which results in a lower current signal (current target). This architecture is defined as signal off, in fact, the higher the target, the lower the current associated. The SPEs that are used and the detection mechanism are reported in Figure 1.

### Electroanalytical measurement

The measurements have been carried out through the use of square wave voltammetry, using the following experimental parameters: E begins of 0 V, E ends of −0.6 V, E steps at 0.001 V, Amplitude at 0.01 V and frequency at 50 Hz. All measurements were carried out using a drop volume of 100 microliters, and all currents were sampled after 30 minutes of the target addition.

## Results and Discussion

### Electrochemical characterization of SPEs

**Figure 2:**
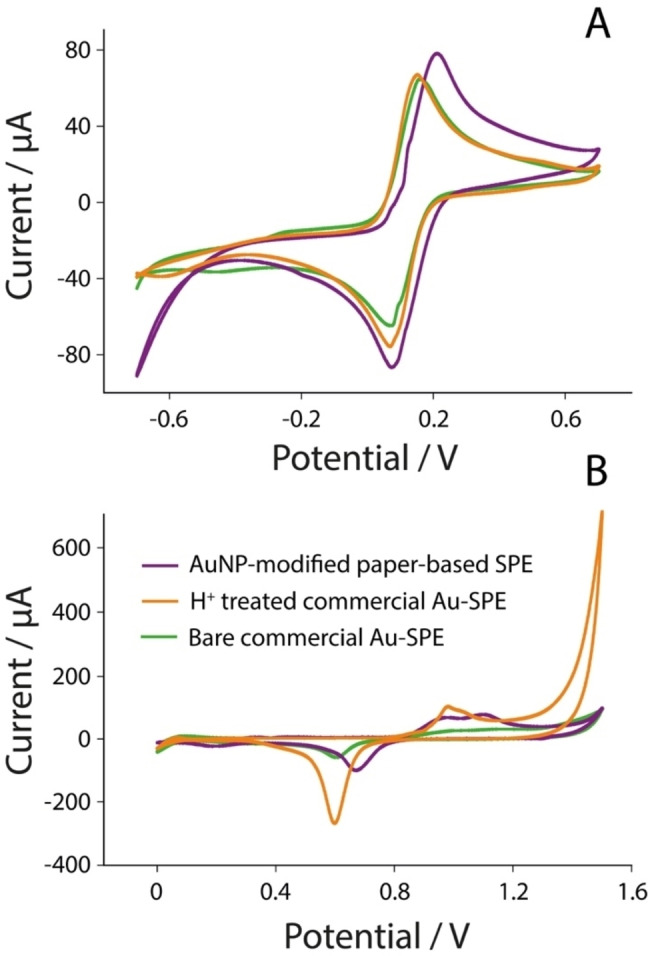
A) Cyclic voltammetry of 5 mM of potassium ferricyanide in 0.1 M KCl with scan rate of 50 mV/s using bare commercial gold‐SPE (green line), acid treated commercial gold‐SPE (orange line) and handmade paper‐based SPE modified with 8 μL of AuNPs (violet line); Cyclic voltammetry of 0.1 M sulfuric acid with scan rate of 0.1 V using bare commercial gold‐SPE (green line), acid treated commercial gold‐SPE (orange line) and handmade paper‐based SPE modified with 8 μL of AuNPs (violet line).

SPEs have been widely applied to analyse many analytes in all existing fields of relevance, such as clinical, environmental, pharmaceutical, agri‐food, etc.[^26^, ^27^, ^28^, ^29^, ^30^, ^31^] However, the choice of the substrate where the SPEs are realised is not always fixed and might depend on the features that are required, i. e. flexibility, mechanical strength, robustness, cost, disposability, etc. For this reason, the first characterisation has focused on the electrochemical evaluation in the presence of an electrochemical mediator, namely potassium ferricyanide, and in the presence of sulfuric acid, as reported in Figure 2.

The three SPE typologies were interrogated in the presence of 5 mM of the electrochemical mediator using a scan rate of 0.05 V/s, as reported in Figure 2A. As can be observed, the shape of the peaks, both the cathodic and anodic ones, confirmed the satisfactory performance of the SPEs in terms of anodic‐to‐cathodic ratio of ca. 1, demonstrating the absence of entrapments at the diverse surfaces, and also experiments carried out by varying the scan rate (up to 0.5 V/s) confirmed that current peaks increased linearly in both cases with the square root of the scan rate, and this is ascribable to a semi‐infinite linear diffusion‐controlled current.[^32^, ^33^, ^34^] In addition to this, as reported in Figure 2B, the three SPEs have been interrogated in presence of 0.1 M sulfuric acid by performing cyclic voltammetry. This experiment allowed us to highlight the gold surface that can be used to consequently attach the thiolated DNA probes. In particular, it should be noted that the appearance of the oxidation peak is ascribable to the formation of oxygen species on top of the gold atoms, while the reduction peak represents the removal of the layer formed by the occurrence of the oxygen species.^[35]^ As displayed in Figure 2B, a difference was observed depending on both the different SPE materials and treatment: in particular, the treated commercial SPE displayed a higher signal with respect to the others, confirming the effect of acidic treatment that is responsible of exposing a higher surface to the working solution. With regard to the paper‐based SPE, modified with AuNPs, the pretreatment was not consistent with an improvement of the electrode performance.

### Optimization of the platforms

**Figure 3:**
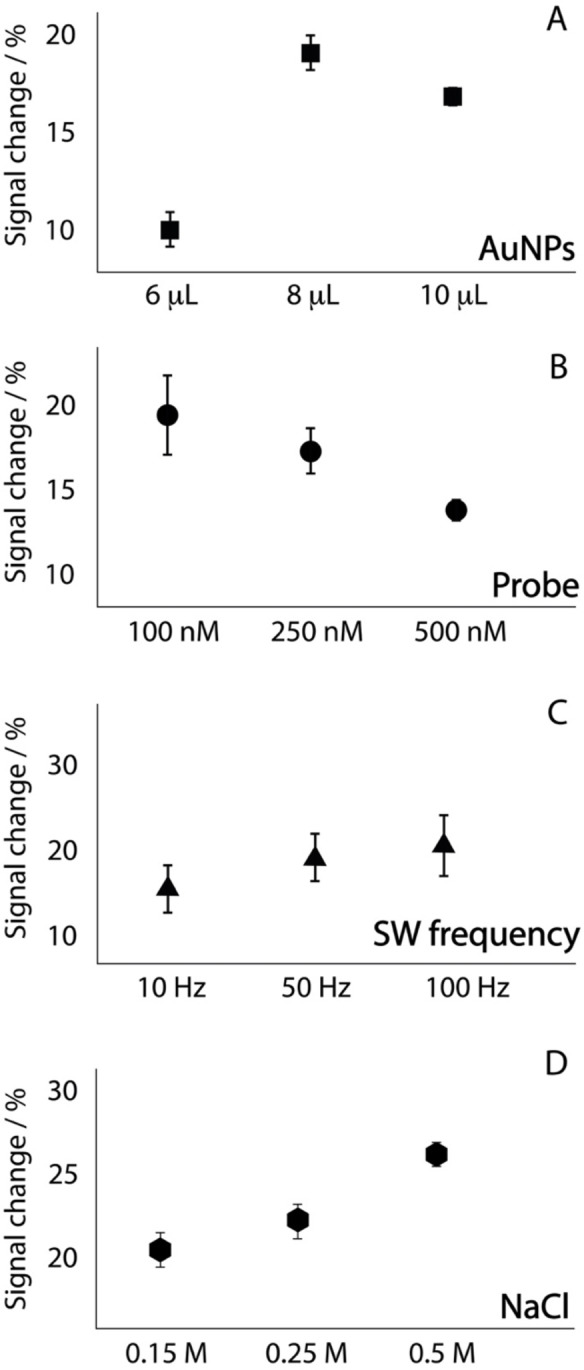
A) Optimization of the amount of microliters of AuNPs drop cast onto the working electrode surface, including 6, 8 and 10 microliters; B) Optimization of the amount of probe to be covalently attached onto the working electrode surface, including 100, 250 and 500 nM of probe solution; C) Optimization of the frequency of the square wave voltammetry to be used to detect the signal due to the MB attached onto the probe, including 25, 50 and 100 Hz; D) Optimization of the concentration of sodium chloride to be used in the working solution, including 0.15, 0.25 and 0.5 M. All the measurements have been carried out in triplicate in 0.05 M phosphate buffer pH 7.4.

In order to obtain high‐performance electrochemical platforms, the most important experimental parameters have been investigated using the paper‐based AuNP‐modified SPE as the model device. In particular, as reported in Figure 3, the amount of AuNPs, the concentration of DNA probe used for the modification of the surface, the frequency of square‐wave voltammetry and the concentration of sodium chloride were tested.

As shown in Figure 3A, the optimal amount of AuNPs was chosen equal to 8 microliters, and this behavior is confirmed by other studies carried out using their modification of the SPE with them. The use of higher amount of nanoparticles was consistent with the decrease of the sensitivity of the device, perhaps due to the formation of less conductive surface as evidenced in previous work.^[36]^ As shown in Figure 3B also the amount of probe to be drop cast on the working electrode was evaluated and 100 nM as the starting solution was chosen as the favorite. The increase of the concentration led to a layer of probes that was too crowded, which limited the affinity toward the target, whereas the lower concentration was consistent with low sensitivity. A previous work reported by the Ricci group demonstrated the effect of probe density on target affinity, highlighting how lower the density of immobilised probes is the higher the affinity toward the density higher is the affinity towards target.^[37]^ Subsequently as shown in Figures 3C and 3D, the frequency of square wave and the concentration of sodium chloride were evaluated, respectively. A frequency of 50 Hz was evaluated as the most suitable for developing the whole platform, producing a satisfactory compromise of signal change and repeatability. With regard to the choice of sodium chloride, the selection of 150 mM as the experimental condition was a consequence of its content in physiological buffer and also because the addition of ulterior salt only produced a slight improvement of signal change.

### Electrochemical detection of miRNA in standard solutions and serum samples

**Figure 4:**
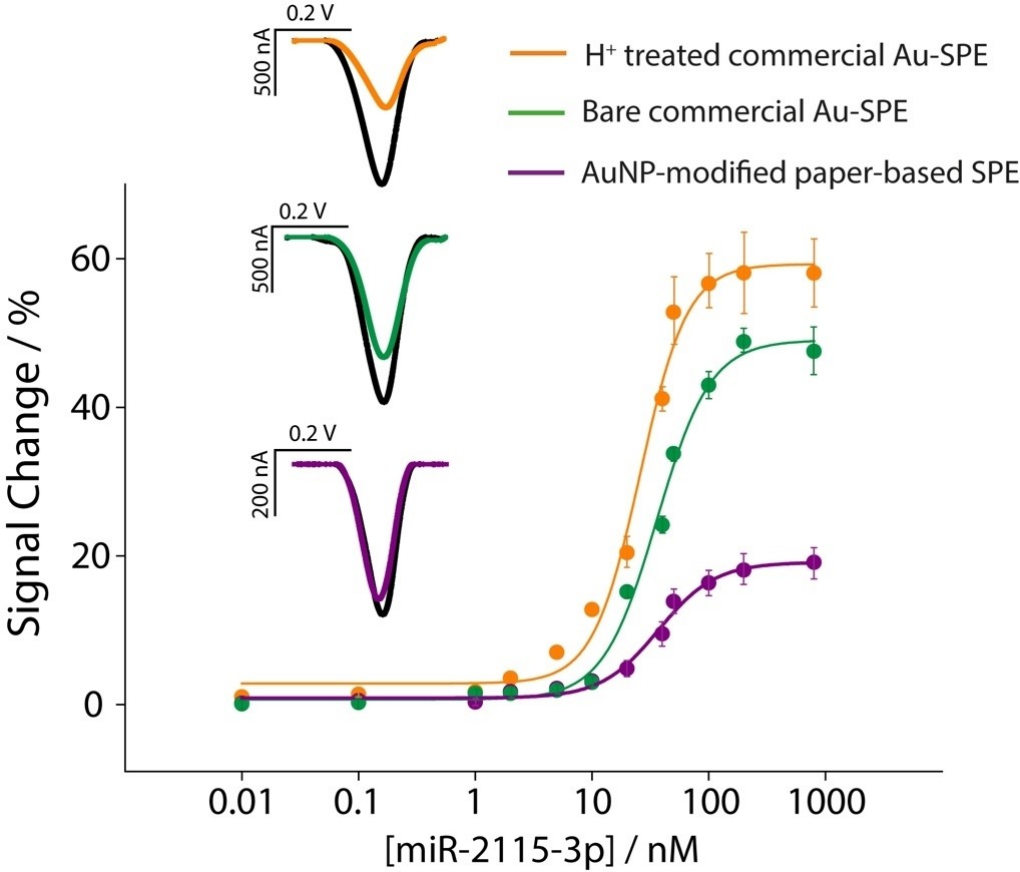
Calibration curves for measurements of miR‐2115‐3p in buffer solution from 0.1 to 1000 nM, using bare commercial gold‐SPE (green line), acid treated commercial gold‐SPE (orange line) and handmade paper‐based SPE modified with 8 μL of AuNPs (violet line). The SW voltammograms are reported in inset, displaying a 100 nM determination of miRNA, comparing the colored lines in the presence of target with the blank reading (black line). All experiments have been carried out in triplicate using the optimal experimental parameters, as discussed in the manuscript.

Following the optimization of key experimental parameters critical to the development of the electrochemical platform, the three SPEs were evaluated for their response to a broad spectrum of miRNA targets, as illustrated in Figure 4.

**Figure 5:**
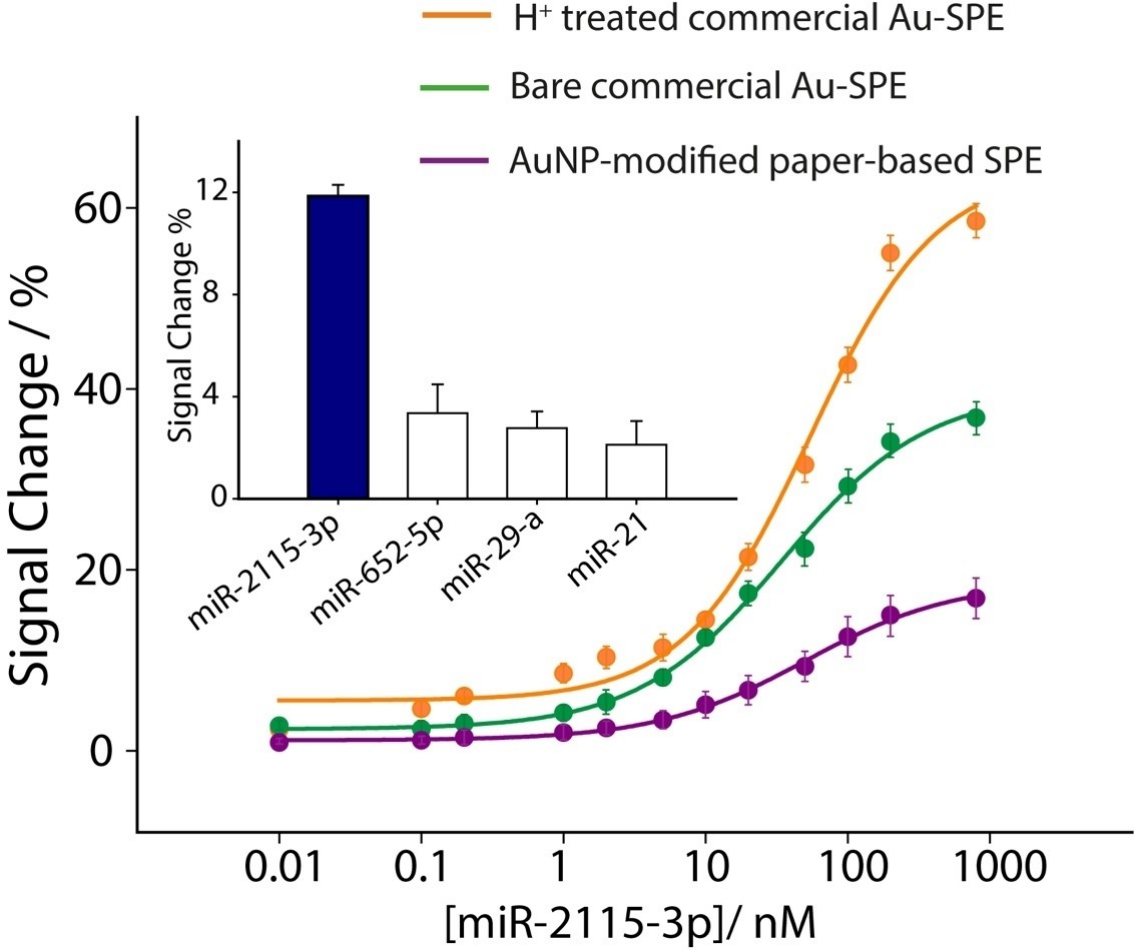
Calibration curves for measurements of miR‐2115‐3p in commercial human serum from 0.1 to 1000 nM, using bare commercial gold‐SPE (green line), acid treated commercial gold‐SPE (orange line) and handmade paper‐based SPE modified with 8 μL of AuNPs (violet line). The SW voltammograms are reported in inset, displaying a 100 nM determination of miRNA, comparing the colored lines in presence of target with the blank reading (black line). Selectivity studies are reported in the inset comparing the signal intensities obtained in the presence of not‐complementary miRNAs. All experiments were carried out in triplicate using the optimal experimental parameters, as discussed in the manuscript.

The three types of SPEs have been interrogated in 0.05 M phosphate buffer, pH 7.4, in presence of 150 mM NaCl, and the concentration of miRNA was varied between a wide range of 0.01 and 1000 nM. All the points that are plotted are the results of three replicates and it should be considered that all the replicates, for all the concentrations tested, have been performed using single shot SPE. What should be observed is how the acidic treatment of the commercial gold‐based SPEs made them the most sensitive platform for miRNA recognition compared to the other two types of SPEs that were taken into account for this study. Even if the signal variation is higher for the commercial gold‐based SPEs, it should be noted how, for all the systems, the first slight variation was detected in a similar range of miRNA. In fact, even if the sensitivity of the linear sections on the fitting curves were different, the detection limits were estimated of ca. 1 nM (the detection limit was calculated as the signal‐to‐noise ratio equal to 3). For all systems, repeatability (calculated as the ration between the standard deviation and the mean values, RSD) was calculated on repetitions using a concentration of 50 nM miRNA, and for all systems tested the RSD never exceeded the 11 %. Subsequently, all systems were challenged in untreated commercial human serum spiked with miRNA in the same range used according to standard solutions, as shown in Figure 5.

As highlighted in Figure 5, the trend of signal variation reported in previous Figure 4 was confirmed and, despite the use of untreated human serum, all SPEs were able to detect the presence of miRNA in the low nanomolar range. These data are in agreement with other reported electroanalytical systems that are based on probe‐target hybridisation as the recognition mechanism of circulating nucleic acids: as reported in our recent review on the use of point‐of‐care testing devices to detect miRNA in biological fluids, the most used voltametric sensors displayed an LOD in the range of 10^−1^–10^0^ nM, while to obtain more sensitive platforms the use of more sophisticated and time‐consuming procedures is usually adopted, i. e. preconcentration, magnetic beads, enzyme amplification.[^15^, ^38^, ^39^, ^40^, ^41^] What it should be taken into account is that the sensitivity of these hybridization‐based sensing devices is majorly ascribable to the probe‐target affinity, that for this kind of systems is around nM range. Moreover, the selectivity of the system has been tested in presence of interfering miRNA sequences, namely 652–5p, 29a and 21, and even a high concentration of 100 nM did not produce significant signal variation (as shown in the inset of Figure 5).

## Conclusions

This manuscript has focused on the evaluation of three different types of SPEs to be used for the detection of circulating miRNA in standard solutions and human serum samples for future application in liquid biopsy. The main goal was to provide the reader with useful information on all the features that should be considered when developing a portable device. In particular, from a sensitivity perspective, the three devices, namely gold‐based commercial SPE, acidic treated gold‐based commercial SPE and AuNP‐modified paper‐based carbon SPE, did not showed significant differences in terms of detection limits in both matrices (within 10^−1^–10^0^ nM) while the binding curves highlighted different slopes in the linear range of signal variations. These differences could be ascribed to the diverse amount of gold on the working electrodes, as confirmed by the cyclic voltametric studies performed in acidic solutions. However, it should be noted that the probe‐target affinity strongly affects the sensitivity of the whole system. In addition, what should be considered when developing this kind of sensing device is related to cost. The cost of one commercial gold‐based SPE is ca. 2–3 Euro, depending on the stock that is purchased, while the entire cost of producing a paper‐based carbon SPE, subsequently modified with few microliters of AuNPs, is around 0.02 Euro.^[42]^

This is something to be taken into consideration, because of the possibility to reduce the production cost by two orders by magnitude, and also to lower the environmental impact related to its disposal (if compared to plastic/ceramic based supports). Novel solutions are strongly required, especially in low/middle income countries where waste disposal is weak, however the only use of paper‐based substrates cannot be enough if all the other components are not sustainable, i. e. water‐based inks should replace the organic‐based ones.^[43]^ Even if the use of paper‐based device is consistent with less robust electrodes, particular attention should be done when modifying, washing and measuring the analyte, with respect to commercial ones. We think that paper‐based electrochemical systems can represent a relevant starting point towards the realization of portable sensing device for liquid biopsy application, even if for the end‐user application the robustness of the commercial SPEs might be considered.

Moreover, with regards the occurrence of miRNA in biofluids, some clarification should be provided the readers. In particular, the detection limit of the present sensing systems are not sufficient (alone) to satisfy the clinical requirement but is should be considered, as reported in literature, all the high performance methods are based on complex architectures. For instance, recently miRNA have been detected down to fM by applying super‐resolution microscopy technique in combination with complex procedures/materials.^[44]^ This is only one of the highest sensitive methods to detect miRNA, however the combination of SPEs with pre‐treatment, magnetic beads, nucleic acid amplification etc. are capable to improve the analytical performance of the presented architecture.

## Conflict of interests

The authors declare no conflict of interest.

1

## Data Availability

The data that support the findings of this study are available from the corresponding author upon reasonable request.
